# Cost-effectiveness analysis of dabigatran and anticoagulation monitoring strategies of vitamin K antagonist

**DOI:** 10.1186/s12913-015-0934-9

**Published:** 2015-07-28

**Authors:** Misericòrdia Carles, Max Brosa, Juan Carlos Souto, Josep Maria Garcia-Alamino, Gordon Guyatt, Pablo Alonso-Coello

**Affiliations:** Departament d’Economia and CREIP, Universitat Rovira i Virgili, Avinguda de la Universitat 1, 43204 Reus, Spain; Oblikue Consulting, Barcelona, SL Spain; Unitat d’Hemostàsia i Trombosi, Hospital de la Santa Creu i Sant Pau, Barcelona, España; Nuffield Department of Primary Care Health Sciences, University of Oxford, Oxford, UK; Department of Clinical Epidemiology & Biostatistics, CLARITY Research Group, McMaster University Medical Centre 2C9, 1200 Main St W, Hamilton, ON Canada; Iberoamerican Cochrane Centre, Biomedical Research Institute Sant Pau (IIB Sant Pau) Research, CIBER of Epidemiology and Public Health (CIBERESP), Sant Antoni M. Claret 167, 08025 Barcelona, Spain

**Keywords:** Atrial fibrillation, Anticoagulant agents, Self-care, Cost an cost-analysis, Drug monitoring

## Abstract

**Background:**

Vitamin K antagonists are commonly used for the prevention of thromboembolic events. Patient self-monitoring of vitamin K antagonists has proved superior to usual care. Dabigatran has been shown, relative to warfarin, to reduce thromboembolic events without increasing bleeding.

**Methods:**

We constructed a Markov model to compare vitamin K self-monitoring strategies to dabigatran including effectiveness and costs of monitoring and complications (thromboembolism and major bleeding). The model was used to project the incidence of these complications, life years, quality-adjusted life years, and health system costs with anticoagulant treatment throughout life. The analysis was conducted from the health system perspective and from the societal perspective.

**Results:**

Low quality evidence suggests that self-monitoring is at least as effective as dabigatran for the outcomes of thrombosis, bleeding and death. Moderate quality evidence that patient self-monitoring is more effective than other forms of monitoring degree of anticoagulation with vitamin K antagonists, reducing the relative risk of thromboembolism by 41 % and death by 34 %. The cost per quality adjusted year gained relative to other warfarin monitoring strategies is well below 30,000 € in the short term, and is a dominant alternative from the fourth year. In comparison with dabigatran, the lower annual cost and its equivalence in terms of effectiveness made self-monitoring the dominant option. These results were confirmed in the probabilistic sensitivity analysis.

**Conclusions:**

We have moderate quality evidence that self-monitoring of vitamin K antagonists is a cost-effective alternative compared with hospital and primary care monitoring, and low quality evidence, compared with dabigatran. Our analyses contrast with the available cost analysis of dabigatran and usual care of anticoagulated patients.

**Electronic supplementary material:**

The online version of this article (doi:10.1186/s12913-015-0934-9) contains supplementary material, which is available to authorized users.

## Background

Continuous oral anticoagulant therapy (OAT) is a common treatment in the primary and secondary prevention of diseases that entail a high risk of thromboembolism. Continuous OAT with vitamin K antagonists (VKAs) is prescribed to approximately 7.2 % of elderly people in developed countries. In Spain, approximately 13.9 of every 1000 people are treated with OAT [1–3].

Continuous therapy with VKAs has serious limitations [4]. In addition to the burden of monitoring, insufficient anticoagulation, carries an increased risk of thrombotic events and excessive anticoagulation an increased risk of bleeding [5–7].

Given the relationship between the international normalized ratio (INR) response and the risk of adverse events, maintaining the patient within the therapeutic range is key when using VKAs [8]. The degree of control is influenced by numerous patient-specific factors, including age, concomitant medications, diet, specific diseases and genetic components. Up to a point, increasing the frequency of testing leads to more results within the therapeutic range [8]. Many factors, including fluctuations in co-morbid conditions, the addition or discontinuation of other medications or changes in diet, may modify desirable testing frequency [8].

The introduction of portable coagulometers (PCs) has allowed the development of alternative control strategies to the standard venopuncture control. These strategies are more accessible to the patient (providing immediate results at their primary care center or at their home), and facilitate an increased frequency of INR monitoring with the possibility of the patient self-adjusting VKA dosing. These devices are as accurate as laboratory machines in measuring the INR [9]. Patient self-management (PSM) strategies have shown superior to usual monitoring of oral anticoagulation [10]. Finally, previous economic evaluations have observed that PSM of VKA therapy, compared to conventional monitoring, is cost-effective [11, 12].

Since 2005, some international consensus and clinical guidelines suggest that PSM [13] is a potential option for patients treated with VKAs who are motivated and can demonstrate competency in self-management strategies, including the self-testing equipment. A recent Spanish clinical practice guideline for the management of atrial fibrillation includes the option of PSM over conventional monitoring [14]. Nevertheless, PSM use is still very limited and the devices and reagents are not yet reimbursed by the National Health System (NHS).

Because, unlike warfarin, the direct thrombin inhibitor dabigatran does not require regular monitoring except in very specific situations, its introduction potentially represents an important advance in OAT. Recently, a large randomized trial, the RELY trial (Randomized Evaluation of Long-Term Anticoagulation Therapy) found that in patients with atrial fibrillation, dabigatran (150 mg) reduced the risk of thromboembolism with risks of bleeding similar to conventionally managed warfarin [15].

At the moment the Spanish Drug Agency recommends the use of VKA, over novel anticoagulants, in already-treated, well-controlled patients, in new patients with non-valvular atrial fibrillation in whom anticoagulant treatment is indicated, and in patients with atrial fibrillation with valvular involvement [16]. This institution does not, however, recommend PSM. Various international [17–19] and local [20] economic analyses have compared dabigatran with conventional control of VKA therapy being generally favorable to dabigatran except in situations of low/moderate risk of thromboembolism or in patients with excellent INR control. However, none of the studies assessed PSM of VKA therapy as an alternative.

The objective of our analysis is two-fold. First, we will determine whether PSM is a cost-effective alternative to conventional monitoring in Spain, taking into account the balance between the cost of PSM and the potential savings derived from a better control (reduction of complications), and the reduction in the costs of supervised INR monitoring in hospitals and primary care centers. We will also determine whether PSM is an efficient alternative to dabigatran.

## Methods

### Design

Cost-effectiveness analysis.

### Population of interest

Patients with conditions that require long term anticoagulation treatment (e.g., atrial fibrillation, mechanical valve diseases or thrombosis) who are candidates for use of both warfarin self-monitoring management strategies and dabigatran.

### Strategies compared

We compared PSM of VKA therapy using a PC with the three strategies currently used in Spain:

- Primary care–PC **(PCpc):** Full monitoring of VKA therapy (extraction of blood samples, interpretation and dose adjustment) by primary care nurses using PC.
- Hospital based anticoagulation clinics–PC **(Hpc):** Conventional monitoring of VKA therapy, with extraction, analysis, interpretation and dose adjustment by hospital specialists using PC.
- Hospital-VP **(Hvp) (dedicated anticoagultion clinic):** Conventional monitoring of VKA therapy, with extraction, analysis, interpretation and dose adjustment by hospital specialists using traditional venopuncture (VP).
- Dabigatran **(Dabi):** treatment with dabigatran etexilate without INR monitoring.

Table 1 describes the characteristics of each of the four options compared. Our analysis assumed that thrombosis and bleeding outcomes were identical in PCpc, Hpc and Hvp; the only differences between the three strategies were in costs. We also assumed that bleeding and thrombosis outcomes were the same in PSM and Dabigatran in the main analysis and in the probabilistic sensitivity analysis, as per our published indirect comparison results.

**Table 1:** Main strategies of oral anticoagulant therapy in Spain

Modality	Test	Dose adjustment	Comments
PSM	Patient	Patient	Dose adjustment may sometimes require telephone help from a health professional
PCpc	RN in primary care center using portable coagulometer	RN in health center	Blood samples may be taken in the patient’s home on some occasions
Hpc	RN in hospital using portable coagulometer	Specialist in hospital	Involves the addition of a portable coagulometer to the conventional centralized model
Hvp	RN in hospital using venipuncture	Specialist in hospital	Conventional centralized mode of OAT control in the so-called “Sintrom Units” in Spain.
Dabi	No monitoring	No adjustment	Dabigatran does not require dose adjustment

*RN* Registered nurse; *PSM* Patient self-management; *PCpc* Primary care using portable coagulometry; *Hpc* Hospital with portable coagulometry; *Hvp* Hospital with venipuncture; *Dabi* Dabigatran

### Type of analysis

**Fig. 1:**
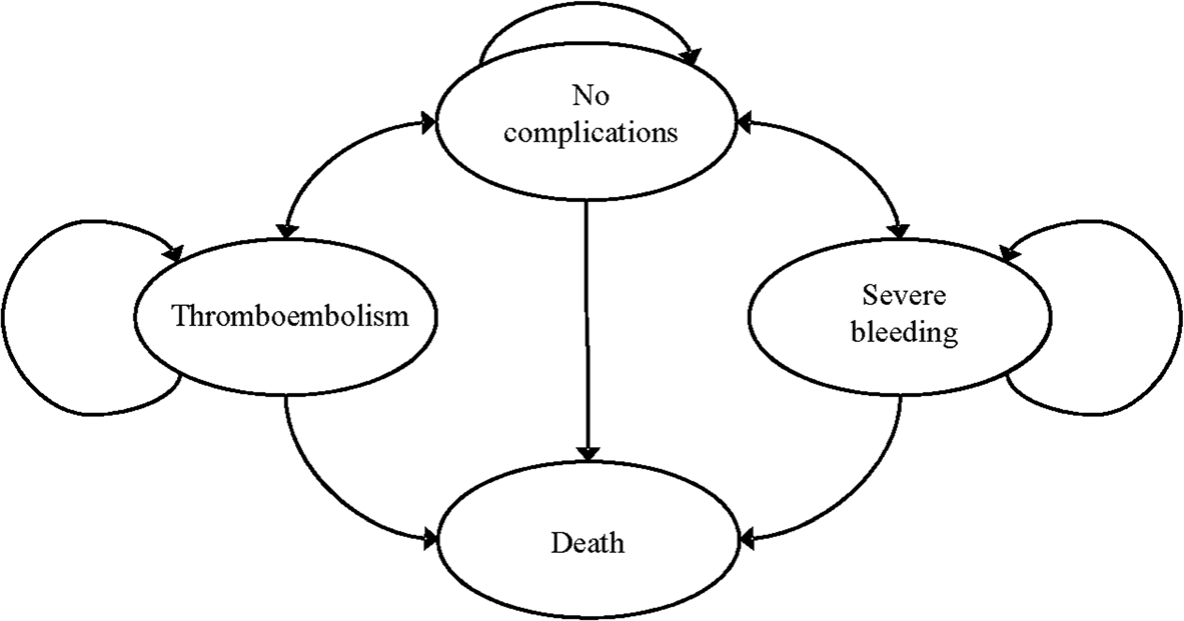
Markov model of OAT

Our cost-effectiveness analysis assessed the incremental costs and effects of PSM vs. other forms of monitoring and dabigatran. Figure 1 shows the schematic Markov model developed to estimate the clinical and economic consequences of the different OAT strategies. Although the lack-of-memory is a property of Markov models, this type of models are especially useful analytical tools in the simulation of chronic health problems and have been used on numerous occasions to estimate costs and effects of interventions that modify the natural history of patients with various diseases. In our model, 1-year Markov cycles were used to represent lifetime outcomes of a cohort of a 67-year old patient.

The following major health states were considered in the Markov model: no complications (where patients remain free of major adverse events), thromboemolism and severe bleeding (with long-term sequelae in 60 % and 10 % respectively [12] and death, as the absorbing Markov state.

### Estimation of health effects

The model draws on data on the incidence of major complications (thromboembolism, major bleeding and death), to represent the evolution of the patients for the different OAT strategies. We obtained the estimates for the comparison of PSM vs conventional monitoring from the Cochrane systematic review published by García Alamino et al. [10]. We obtained the estimates for the PSM vs dabigatran comparison from an indirect analysis of PSM with dabigatran [21]. The overall quality of the evidence according to the GRADE system [22] for the direct comparison is moderate (due to risk of bias and imprecision) and low for the indirect comparison (due to risk of bias, indirectness and imprecision). Table 2 summarizes the clinical parameters and utility values used in our model [23], which together allowed us to estimate both life years gained (LYG) and quality adjusted life years (QALY) associated to compared options.

**Table 2:** Clinical parameters of the model (annual rates of complications)

	Thromboembolism	Hemorrhage	Death
Base analysis (deterministic)			
Annual rate Hvp	0.052	0.079	0.093
RR PSM vs Hvp	0.59 (0.46–0.77)	1	0.76 (0.58–0.99)
RR Dabi vs Hvp	0.59 (0.46–0.77)	1	0.76 (0.58–0.99)
RR PCpc vs Hvp	1	1	1
Base analysis (probabilistic)			
Annual rate Hvp	0.052	0.079	0.093
RR PSM vs Hvp	0.59 (0.46–0.77)	0.96 (0.81–1.13)	0.76 (0.58–0.99)
RR Dabi vs Hvp	0.66 (0.53–0.82)	0.93 (0.81–1.07)	0.88 (0.77–1.00)
Other variables			
% of non-fatal complications that cause permanent disability	60 %	10 %	-
% of patients with permanent disability who continue therapy	50 %	50 %	-
Utility without complications	0.659	0.659	-
Utility with complication	0.447	0.215	-

*RR* Relative risk; *PSM* Patient self-management; *PCpc* Primary care with portable coagulometer; *Hpc* Hospital with portable coagulometer; *Hvp* Hospital with venipuncture; Dabi: Dabigatran

Sources: adapted from Brown A. et al. (2007) [12], Alonso-Coello, P et al. [21, 37]

### Estimated impact on resources (quantification and measurement)

To calculate the economic consequences of various options, we estimated the health and non-health (time of patient and companion, and travel) resources used according to the results of a previous Spanish technology assessment [3] and expert opinion (Table 3). We assumed that OAT with dabigatran does not require INR monitoring, but did require a specialist visit for patient monitoring.

**Table 3:** Use of health resources in monitoring of OAT

	First year					Successive years				
	PSM	PCpc	Hpc	Hvp	Dabi	PSM	PCpc	Hpc	Hvp	Dabi
Health costs										
N° of checkups/year	52	13	13	13	-	52	13	13	13	-
Specialist (min/control)			2	2				2	2	
Nurse (min/control)		5	5	5			5	5	5	
Test (venous blood/control)				1					1	
Test (Coaguchek strips/control)	1	1	1			1	1	1		
Nurse (min/training)	240	20	20	20	20		-	-	-	-
Non health costs										
Patient time^a^ (min/control)	5	60	120	120	-	5	60	120	120	-
Companion time^a^ (min/control)	5	60	120	120	-	5	60	120	120	-
Patient time (min/training)	240	-	-	-	-	-	-	-	-	-
Companion time (min/training)	240	-	-	-	-	-	-	-	-	-
% of patients with companion	23.4 %	25.6 %	25.6 %	25.6 %	-	23.4 %	25.6 %	25.6 %	25.6 %	-

^a^Assuming a mean trip of 2 km and 30 km for control in primary care and hospital, respectively

*PSM* Patient self-management; *PCpc* Primary care with portable coagulometer; *Hpc* Hospital with portable coagulometer; *Hvp* Hospital with venipuncture; *Dabi* Dabigatran

Unit costs were applied to each of the resources measured. The product of the amount of resources used (drug devices, test strips, clinicians’ time, consumables, etc.) times the unit cost provided the health costs of the options studied. We also calculated the costs of complications. The costs of thromboembolism were calculated using the weighted mean cost of DRG (Diagnostic Related Groups categories) codes for stroke, transient ischemic attack and pulmonary embolism obtained from the latest dataset of the Minimum Data Set of the Spanish National Health System (MSC 2010) and 3-year stroke costs from a Spanish retrospective study [24], while the cost of severe bleeding was calculated using the mean cost of two (DRG) (DRG 174 and 175–Gastrointestinal bleeding with and complications respectively) included in the latest data of the Minimum Data Set of the Spanish National Health System [25]. Table 4 shows unit costs of drugs and monitoring and the costs associated with each complication of OAT.

**Table 4:** Unit costs of INR monitoring and drugs

Unit costs	Cost (€ 2012)
Daily cost acenocumarol	0.13 €
Daily cost dabigatran	3.03 €
Hour of specialist time	59.9 €
Hour of nursing time	15.8 €
Hour of family physician time	26.7 €
Venipuncture (syringe, tube,.,)	0.5 €
Reactive strip Coaguchek	2.7 €
Coagulometer (Coaguchek)^a^	588.5 €
Thromboembolism: first year	6556 €
Thromboembolism: successive years	4470 €
Severe bleeding: first year	3135 €
Severe bleeding: successive years	0 €
Cost per hour patient/companion	17.3 €
Cost per km. of travel	0.51 €

^a^Assuming offsetting of 5 years for each device; assuming the use of one PC for each 10 patients monitored in the case of primary care and hospitals

Source: website of Ministerio de Sanidad, Servicios Sociales e Igualdad (http://www.msssi.gob.es/estadEstudios/estadisticas/cmbd/informes/home.htm) and Oblikue Consulting eSalud health database (http://www.oblikue.com/bddcostes/)

### Perspective, time horizon and discount

The analysis was conducted from the perspective of the Spanish NHS (including only direct health costs). We also conducted an analysis from the societal perspective including costs of caregivers’ time. Methods for this analysis followed the approaches used by similar published analysis in the field [12, 26, 27]. This perspective is included because these costs can have a significant effect on cost-effectiveness analysis and can vary among anticoagulation approaches. The time horizon was from one year to the lifetime of the patient. Both the costs and effects were discounted using an annual rate of 3 %.

### Sensitivity analysis

We carried out a univariate sensitivity analysis to observe the individual influence of key parameters on results. The variables included in this analysis where: the relative risk of developing a thrombotic complication, the relative risk of developing a severe haemorrhage complication and relative risk of death, proportion of permanent complications, proportion of treatment discontinuation in patients with permanent complications, utility values, monitoring costs and complications costs Additionally, we performed a probabilistic sensitivity analysis according to the most relevant recommendations in the health technology assessment field [28, 29]. The main advantage of this type of analysis, which uses the Monte-Carlo simulation technique, is that several model parameters can be varied simultaneously, allowing the variability of the data and the expected results to be reflected better. Specifically, we used a beta distribution for the probabilities and utilities of the model and a log-normal distribution for the costs.

## Results

**Fig. 2:**
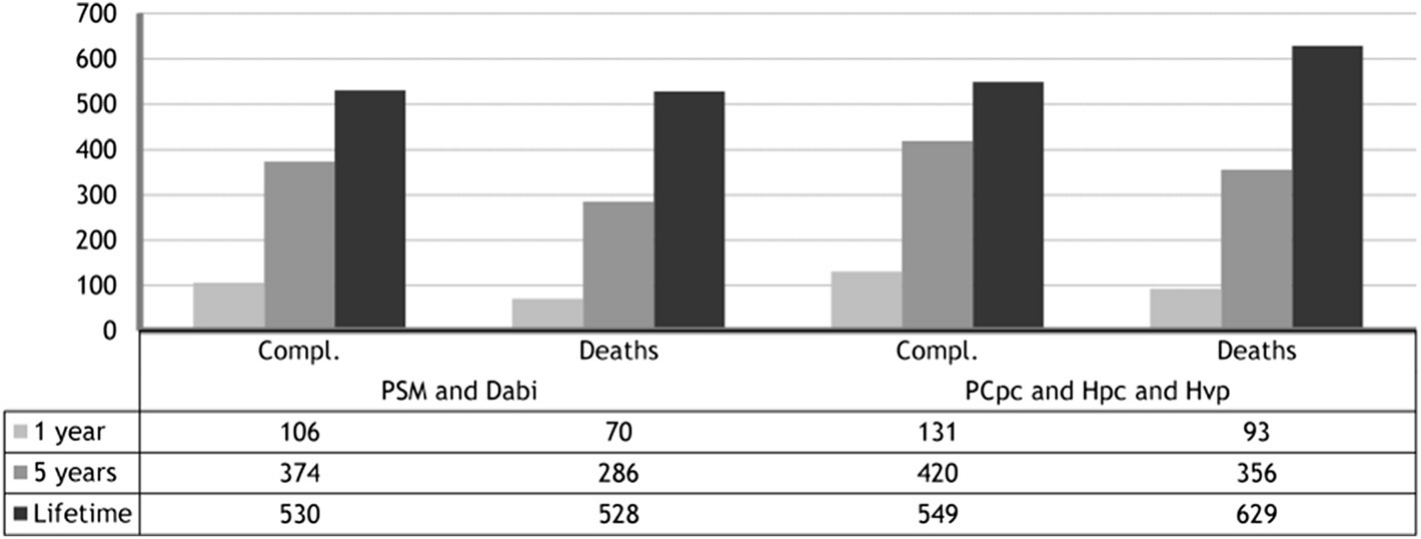
Compl: Complications (thromboembolism and severe bleeding); PSM: Patient self-management; PCpc: Primary care with portable coagulometer; Hpc: Hospital with portable coagulometer; Hvp: Hospital with venipuncture. Dabi: Dabigatran

Figure 2 shows the number of complications (thromboembolism and severe bleeding) and deaths per 1000 patients controlled by the different options, for three time horizons (1 year, 5 years and the patient’s lifetime).

Table 5 shows survival and quality-adjusted survival of the options analyzed in terms of the time horizon of the analysis. Given our assumptions about the effectiveness of the proposed options, we compare PSM and Dabigatran with VKA therapy monitored in the hospital (Hvp and Hpc) and primary care (PCpc), the first alternative being more effective than the second. The lower incidence of complications for PSM and Dabigatran translates into a 1.16 life years gain and a 0.6 QALY gain in the long-term analysis.

**Table 5:** Basic results. LY and QALY of the options compared

	Life years		Quality-adjusted life years	
Years since initiation	PSM and Dabi	PCpc and Hpc and Hvp	PSM and Dabi	PCpc and Hpc and Hvp
*1*	*0.90*	*0.88*	*0.56*	*0.54*
*5*	*3.77*	*3.55*	*2.21*	*2.06*
10	6.09	5.51	3.43	3.10
*Lifetime*	*8.45*	*7.29*	*4.59*	*3.99*

*PSM* Patient self-management; *PCpc* Primary care with portable coagulometer; *Hpc* Hospital care with portable coagulometer; *Hvp* Hospital care with venipuncture; *Dabi* Dabigatran

Table 6 shows the basic results of the analysis in terms of cost (monitoring, complications and total) per patient for various periods of follow up of up to 10 years and for the patient’s lifetime. Dabigatran had the highest cost for the entire period, followed by PSM, although the differences between PSM and conventional hospital care (Hpc, Hvp) and primary care (PCpc) decreased so that from the second year (vs Hpc) or the third and fourth years (vs Hvp and PCpc, respectively) onwards PSM was the least costly option. This decrease in the total cost of PSM was due to lower costs associated with thromboembolism and major bleeding, which had a higher incidence in other conventional forms of OAT. When non-healthcare costs were included, the savings associated with PSM option became evident from the second year (vs PCpc) and the first year vs. the other options.

**Table 6:** Basic results. Disaggregated costs (€) of the options compared

Years since initiation	PSM			PCpc			Hpc			Hvp			Dabi		
	Total	AC	Compl	Total	AC	Compl	Total	AC	Compl	Total	AC	Compl	Total	AC	Compl
Spanish National Health Service Perspective															
1	1066	659	407	820	259	562	967	409	558	884	324	560	1454	1058	396
5	3692	1373	2319	3846	644	3202	4296	1124	3173	4148	969	3179	5757	3631	2125
10	6217	1747	4470	6707	800	5907	7269	1415	5854	7096	1231	5865	9026	4981	4045
Lifetime	9118	1938	7179	9668	858	8810	10,266	1521	8745	10,084	1327	8756	12,198	5672	6526
Societal perspective															
1	1228	826	402	1081	526	555	1640	1100	540	1557	1015	542	1454	1058	396
5	4059	1755	2305	4648	1501	3147	6367	3339	3029	6221	3184	3037	5757	3631	2125
10	6691	2241	4450	7706	1898	5808	9846	4250	5596	9675	4067	5609	9026	4981	4045
Lifetime	9653	2491	7162	10,728	2043	8685	12,999	4583	8416	12,821	4389	8432	12,198	5672	6526

*AC* Anticoagulation including INR monitoring plus drug cost; *PSM* Patient self-management; *PCpc* Primary care with portable coagulometer; *Hpc* Hospital care with portable coagulometer; *Hvp* Hospital care with venipuncture; *Dabi* Dabigatran; *Compl* complications

Table 7 shows the results of cost-effectiveness analysis, and demonstrates that PSM had ratios for Cost/QALYs gained far below the € 30,000 considered as the cost-effectiveness threshold in Spain [30] from the first year of follow up onwards, and was the dominant option, with greater effectiveness and lower costs than the other conventional options from the fourth year of follow up onwards. In comparison with dabigatran, the lower annual cost (from the first year of follow-up) and its equivalence in terms of effectiveness made PSM the dominant option. From the societal perspective, PSM was dominant from the first year in all cases (except vs PCpc, in which it was dominant from the second year).

**Table 7:** Basic results. Cost-effectiveness analysis (Cost/QALY gained)^a^

Years since initiation	PSM vs PC	PSM vs Hpc	PSM vs Hvp	PSM vs Dabi
1	12,289 €	4960 €	9120 €	Dominant
2	3519 €	Dominant	275 €	Dominant
3	770 €	Dominant	Dominant	Dominant
4	Dominant	Dominant	Dominant	Dominant
5	Dominant	Dominant	Dominant	Dominant
10	Dominant	Dominant	Dominant	Dominant
Lifetime	Dominant	Dominant	Dominant	Dominant

*PSM* Patient self-management; *PCpc* Primary care with portable coagulometer; *Hpc* Hospital with portable coagulometer; *Hvp* Hospital with venipuncture; *Dabi* Dabigatran

^a^The basic results are shown from the perspective of the health system. In the analysis from the social perspective, PSM was the dominant option in all cases except for the analysis at 1 year vs. PCpc, in which the iCER was 7352 € per QALY gained

### Results of the sensitivity analysis

The results of the one-way sensitivity analysis showed that PSM was a dominant option vs all comparators when using extreme values of the 95 % CI of relative risks of complications and death (except for the upper value of the RR of thrombotic event, when PSM was dominant against all options but PCpc, presenting a cost per QALY of 1945€), varying ±25 % the proportion of permanent complications, the proportion of treatment discontinuation in patients with permanent complications and utility values. Varying ±25 % monitoring + drug costs and complications costs yielded to the same results, i.e., PSM dominates all other comparators, except when monitoring costs for PSM where assumed to be 25 % higher than those in the base case (when this was assumed, PSM dominated all options except PCpc, with a cost per QALY < 1000 €).

Additional file 1: Figure S3, Additional file 2: Figure S4, Additional file 3: Figure S5, Additional file 4: Figure S6 and Additional file 5: Figure S7 show the results of the sensitivity analysis. That is, the results of the probabilistic cost-effectiveness analysis of PSM vs. each of the alternative options in the medium term (five years). This analysis underlines the robustness of the results, showing that PSM is the dominant option in all four comparisons, i.e., with lower costs and similar effectiveness (vs. dabigatran) or greater effectiveness (vs PCpc, Hpc and Hvp) in 78 %, 59 %, 84 % and 77 % of the simulations. In the remaining simulations, the cost per QALY gained for PSM was below the € 30,000 reference threshold in Spain.

## Discussion

This study demonstrates that on the basis of existing limited evidence, PSM is a more cost-effective OAT strategy than neither alternative monitoring systems of anticoagulation or dabigatran. Low quality evidence suggests similar effectiveness to that of the first oral anticoagulant that does not require INR monitoring (dabigatran); moderate quality evidence indicates that PSM has greater effectiveness than all the options of monitoring of VKA therapy. PSM has, over the long term, lower costs than all the alternatives.

Since 1999, a number of economic analyses have examined the efficiency of different control strategies of VKA therapy [11, 12, 31–34]. All but one study recommended, firstly, the use of PC techniques over traditional venopuncture and laboratory analysis, due both to the costs of laboratory analysis and to the greater accessibility and rapidity of PC. Even the study by Connock et al. [34] highlighted the positive influence on the quality of life, patients’ to undertake self-management of therapy and the reduced adverse effects of treatment with PC. The results concerning the optimal alternative strategy–who should do the testing and where should it be done (dedicated anticoagulant clinic often located in a hospital, primary care or PSM) are more variable. First, as in our study, all analyses found that strategies involving greater patient participation are more costly in terms of direct health resources (devices, frequency of controls, reagents, etc.), but reduce the costs for the patient, although few studies have measured this aspect. The increase in direct costs seems logical since the very purpose of these strategies is to increase the frequency of monitoring, but the absolute increase depends largely on the organization of the program, the training mechanisms and the support staff considered.

The economic evaluations conducted also found, as did our study that increas[es in the direct costs of strategies involving greater patient participation were compensated for in the medium- and long-term by the benefits (costs avoided) derived from these strategies, the reduction in the incidence of complications. Thus, in studies in which the incidence of complications in the long term is estimated according to the percentage of time in the therapeutic range, PSM alternatives dominated the rest (i.e., had a lower cost and greater effectiveness).

In the only two economic studies directly related to clinical trials [27, 35], in which clinical outcomes were observed only in the short term, the differences found in effectiveness were not big enough to compensate for the higher direct short-term costs of PSM strategies. However, the longer time horizon of studies using models or the time of follow up in specific prospective studies improves the results in favor of PSM, as they allow for recouping costs in the medium-term and increasing the differences found in the incidence rates of adverse events. Another factor in the studies analyzed that improves the outcomes of PSM strategies, particularly in organizational models that include patient support units, is the number of patients included. Increases in patient volume largely compensate for some of the fixed costs associated with the program.

In short, the results obtained in our study for this comparison are similar to those of other studies using modeling in which the benefits of control techniques are estimated according to the percentage of time within the therapeutic range or the incidence of complications. PSM strategies (either monitoring or monitoring and adjustment) are more effective than other modalities of VKA monitoring in patients with similar characteristics, because, by avoiding complications, they reduce costs, thus offsetting the increase in resources used for monitoring. Savings amount to more than €500 per patient during the patient’s lifetime. In our analysis, PSM was a dominant alternative compared to other methods of VKA monitoring (it had lower total costs and greater effectiveness), from the fourth year of follow up.

With respect to the efficiency of the new oral anticoagulants, dabigatran has been shown internationally to be cost-effective for thromboembolism prevention in populations mainly at high risk of stroke or with suboptimal INR control [17–19]. The incremental cost-effectiveness ratio of dabigatran was below €32,000 over a 10-year time horizon compared to conventional OAT in a recent Spanish study [20]. However, none of the above analyses specifically compared dabigatran with the option of PSM of conventional OAT. In our study, using the results of an indirect comparison of PSM vs. dabigatran, PSM was shown to be more efficient with lower health costs than dabigatran from the first year of follow up [21].

Our analysis has several limitations. Firstly, the quality of the evidence from the indirect comparison of PSM respect to dabigatran is low, due to risk of bias, imprecision and indirectness [21]. Indirectness is a particular concern because of differences in populations enrolled in the self-monitoring trials and the dabigatran trial. To begin with, all patients in the dabigatran trial had atrial fibrillation, while the patients in the self-monitoring had a variety of conditions requiring anticoagulation. Not all patients – and possibly only a minority of patients – in the dabigatran trial would have been candidates for self-monitoring. Patients excluded in the dabigatran trial because of renal dysfunction would have been enrolled in the warfarin self-monitoring trials [13, 36]. On the other hand, there are sufficient similarities in the populations that the indirect comparison warrants attention. The dabigatran and home monitoring studies enrolled patients of similar age, received similar co-interventions, measured outcomes in similar ways, achieved similar rates of follow-up and had similarly low risk of bias. Most important, anticoagulant control measured by TTR was similar in the conventional warfarin arm of RELY (64 %) and the home-monitoring studies (61.9 %) [10, 15].

Although the greatest limitation of PSM is its applicability (approximately 25–40 % of patients with VKA are candidates for PSM) [13], our results suggest that for those for whom it is appropriate, PSM is superior to conventional OAT monitoring and may also be superior to dabigatran. These results are likely to have important implications for the Spanish national health system and elsewhere. Another limitation is that the model did not include the possibility that patients may switch between different options, but this assumption was made to better analyse the differences between options, even no conclusions may be done regarding strategies that describe sequential treatments and monitoring modalities. Finally, in keeping with previous economic analysis [31–34], we did not include productivity cost of patients in the societal perspective analysis. However, because the population of interest is mostly over 65 years of age, inclusion of such costs would be unlikely to have a major influence on the results.

The strengths of our analysis include use of the best available evidence on the effectiveness of compared options including our recently published indirect comparison of PSM and dabigatran. The inclusion of non-medical costs is particularly important in this condition, and patient’s time necessary to carry out INR monitoring might be reduced with PSM despite the increase in frequency of tests.

A final point has to do with individualization of choice of therapy. Although individualized decision-making has not been formally tested, it is a standard part of clinical practice. PSM is only applicable to those who are interested and capable. Even among those potentially interested and capable, there may be patients who place a very high value on reducing burdens associated with medication use. Whatever the cost implications, such patients would likely be best served by using dabigatran for anticoagulation.

## Conclusions

In summary, the available evidence suggests that PSM of VKAs is a cost-effective alternative compared with hospital and primary care monitoring, and also compared with dabigatran. However, the confidence in the estimates is low. Our analyses contrast with the available cost analysis of dabigatran with PSM.

## Additional files

Additional file 1: Figure S3. Cost-effectiveness of patient self-management vs. primary care. Additional file 2: Figure S4. Cost-effectiveness of patient self-management vs. hospital care (portable coagulometer). Additional file 3: Figure S5. Cost-effectiveness of patient self-management vs. hospital care (venopucture). Additional file 4: Figure S6. Cost-effectiveness of patient self-management vs. dabigatran. Additional file 5: Figure S7. Cost-effectiveness acceptability curve (CEAC).

## Abbreviations

PCpc: Primary care – portable coagulometers
Dabi: Dabigatran
DRG: Diagnostic Related Groups categories
Hvp: Hospital-VP
INR: International normalized ratio
LYG: Life years gained
MSC: Spanish National Health System
NHS: National Health System
OAT: Continuous oral anticoagulant therapy
PCs: Portable coagulometers
PSM: Patient self-management
QALY: Quality adjusted life years
RELY: Randomized Evaluation of Long-Term Anticoagulation Therapy
VKAs: Vitamin K antagonists
VP: Venopuncture
