# Supplementary figures and images for: Cost-effectiveness analysis of dabigatran and anticoagulation monitoring strategies of vitamin K antagonist
Source: BMC Health Serv Res. 2015 Jul 28;15:289. doi: 10.1186/s12913-015-0934-9 (PMC4515878; doi:10.1186/s12913-015-0934-9)

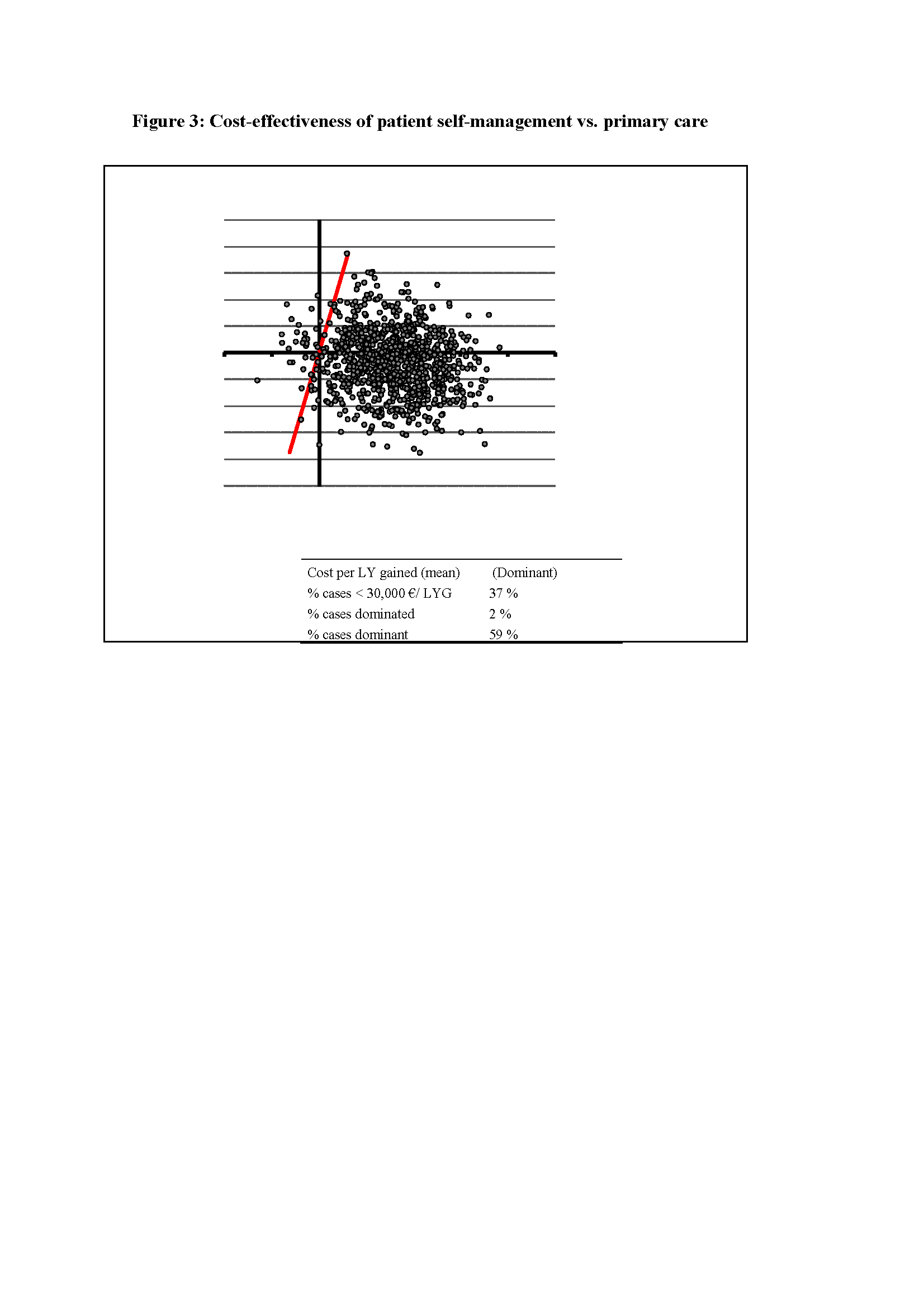

Supplement: Additional file 1: Figure S3. — Cost-effectiveness of patient self-management vs. primary care. [file 12913_2015_934_MOESM1_ESM.tiff]

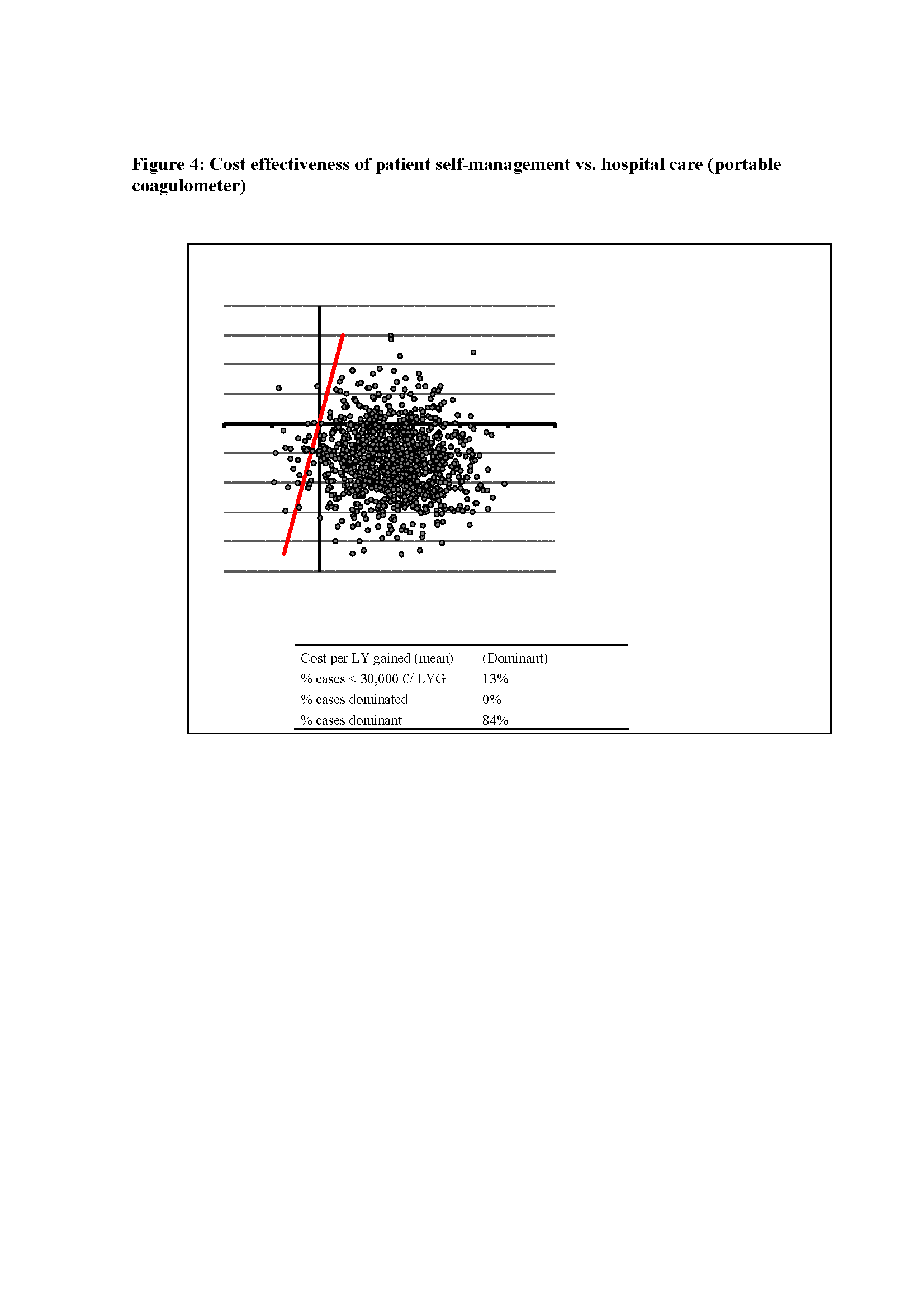

Supplement: Additional file 2: Figure S4. — Cost-effectiveness of patient self-management vs. hospital care (portable coagulometer). [file 12913_2015_934_MOESM2_ESM.tiff]

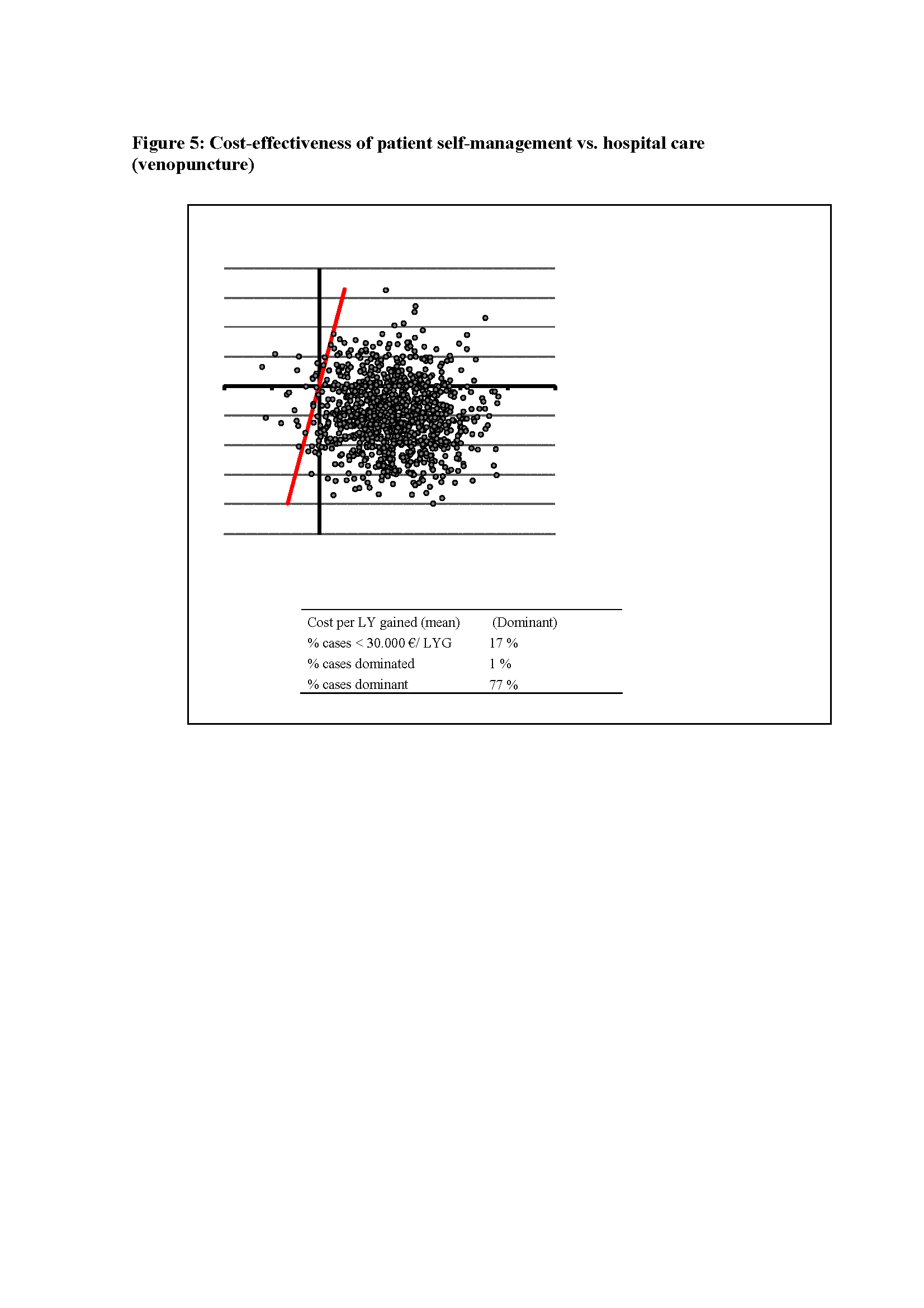

Supplement: Additional file 3: Figure S5. — Cost-effectiveness of patient self-management vs. hospital care (venopucture). [file 12913_2015_934_MOESM3_ESM.tiff]

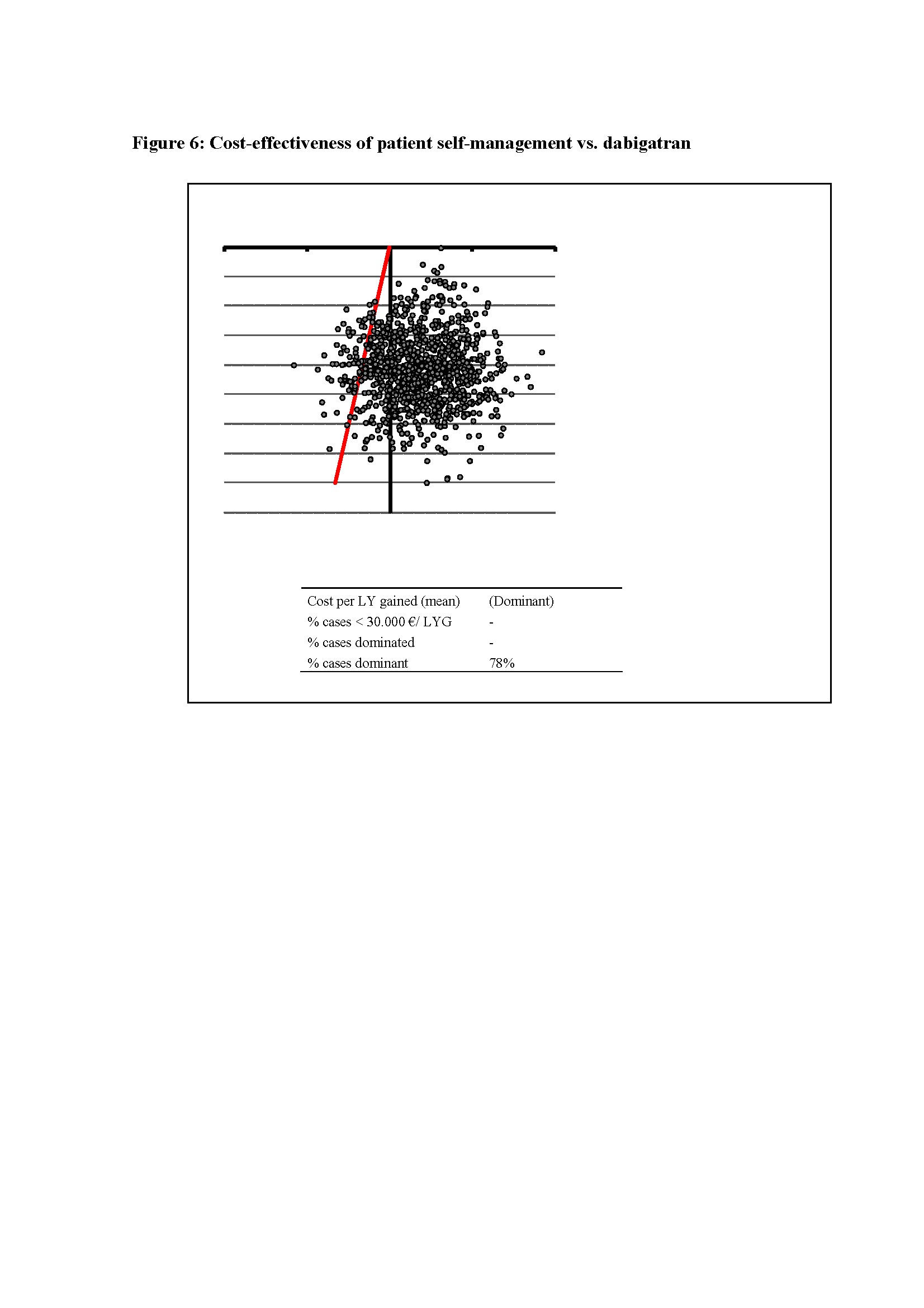

Supplement: Additional file 4: Figure S6. — Cost-effectiveness of patient self-management vs. dabigatran. [file 12913_2015_934_MOESM4_ESM.tiff]

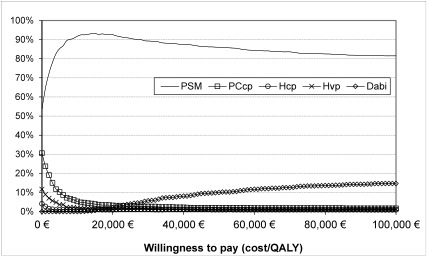

Supplement: Additional file 5: Figure S7. — Cost-effectiveness acceptability curve (CEAC). [file 12913_2015_934_MOESM5_ESM.jpeg]
